# Smartphone Applications to Support Tuberculosis Prevention and Treatment: Review and Evaluation

**DOI:** 10.2196/mhealth.5022

**Published:** 2016-05-13

**Authors:** Sarah J Iribarren, Rebecca Schnall, Patricia W Stone, Alex Carballo-Diéguez

**Affiliations:** ^1^ Columbia University School of Nursing New York, NY United States; ^2^ New York State Psychiatric Institute and Columbia University Department of Psychiatry New York, NY United States

**Keywords:** mobile apps, mobile health, tuberculosis, review

## Abstract

**Background:**

Tuberculosis (TB) remains a major global health problem and is the leading killer due to a single infectious disease. Mobile health (mHealth)–based tools such as smartphone apps have been suggested as tools to support TB control efforts (eg, identification, contact tracing, case management including patient support).

**Objective:**

The purpose of this review was to identify and assess the functionalities of mobile apps focused on prevention and treatment of TB.

**Methods:**

We searched 3 online mobile app stores. Apps were included if they were focused on TB and were in English, Spanish, or Portuguese. For each included app, 11 functionalities were assessed (eg, inform, instruct, record), and searches were conducted to identify peer-review publications of rigorous testing of the available apps.

**Results:**

A total of 1332 potentially relevant apps were identified, with 24 meeting our inclusion criteria. All of the apps were free to download, but 7 required login and password and were developed for specific clinics, regional sites, or research studies. Targeted users were mainly clinicians (n=17); few (n=4) apps were patient focused. Most apps (n=17) had 4 or fewer functions out of 11 (range 1-6). The most common functionalities were inform and record (n=15). Although a number of apps were identified with various functionalities to support TB efforts, some had issues such as incorrect spelling and grammar, inconsistent responses to data entry, problems with crashing, or links to features that had no data. Of more concern, some apps provided potentially harmful information to patients, such as links to natural remedies for TB and natural healers. One-third of the apps (8/24) had not been updated for more than a year and may no longer be supported. Peer-reviewed publications were identified for only two of the included apps. In the gray literature (not found in the app stores), three TB-related apps were identified as in progress, being launched, or tested.

**Conclusions:**

Apps identified for TB prevention and treatment had minimal functionality, primarily targeted frontline health care workers, and focused on TB information (eg, general information, guidelines, and news) or data collection (eg, replace paper-based notification or tracking). Few apps were developed for use by patients and none were developed to support TB patient involvement and management in their care (eg, follow-up alerts/reminders, side effects monitoring) or improve interaction with their health care providers, limiting the potential of these apps to facilitate patient-centered care. Our evaluation shows that more refined work is needed to be done in the area of apps to support patients with active TB. Involving TB patients in treatment in the design of these apps is recommended.

## Introduction

Tuberculosis (TB) remains a major global health problem and is the leading killer due to a single infectious disease. The World Health Organization (WHO) estimates that one-third of the world population harbors latent TB infections, 14.1 million people have active cases, 9 million are newly diagnosed per year, and 1.5 million deaths are attributable to TB annually [1-3]. This death toll equals 2% of global mortality, yet it is a disease for which a cure has existed for 70 years. Given that most deaths from TB are preventable, this death toll is recognized by WHO as unacceptably high [1].

A number of factors contribute to the persistence of TB and its high mortality rate. For example, factors that have been identified as impacting TB medication adherence include length of treatment course, complex regimens, medication side effects, poor access to health care services, poor communication with providers, lack of social support, negative perceptions, and stigma and discrimination [4]. Directly observed therapy (DOT), where a trained health care worker or treatment supporter observes medication ingestion daily, is a WHO-recommended strategy to provide patient support and assure drug adherence for TB treatment. However, there are challenges to implementing DOT in many settings [5]. For instance, DOT is labor-intensive, transportation-dependent, and often inconvenient; therefore, in some regions TB treatment is offered by self-administration when attention from health care workers is unavailable, costly, or difficult to access due to geographic distances [6,7]. No matter how the treatment is given, heightening patients’ involvement in their own care; improving communication between patients and health care teams; and providing flexible patient-centered care, education, and support to patients during treatment are recommended to increase TB medication adherence [4,7].

There has been a rapid development of computers and information technology in all aspects of life including health care over the past 15 years [8]; one type of health care information technology development is mobile health (mHealth). Smartphone apps are reported to be ideal platforms for improving health outcomes because of their popularity, connectivity, and sophistication [9]. Increasingly, apps can support added functionalities beyond, for example, text messaging and have the potential for real-time data collection, graphic feedback, interactivity, and links to social functionalities. Apps are being developed as tools to support multiple aspects of health care, including prevention, diagnosis, data collection, treatment adherence monitoring, and disease surveillance. Apps have the potential to support TB prevention and treatment efforts by supporting health care providers in diagnosing TB, monitoring patient progress, and providing support to patients to successfully complete treatment [6].

In the past few years, the number of health-related apps available to consumers has more than doubled for the two leading platforms, iOS and Android [10]. A study published in 2015 by the IMS Institute for Health care Informatics identified more than 165,000 health-related apps [11] compared to about 40,000 in its 2013 report [12]. While there is a large literature base on human-computer interaction in many areas [13-15], the methods for review and assessment of health-related apps are evolving. Prior reviews report systematic methods for conducting searches in the literature and app stores to describe the breadth of apps available [16,17], while others have added detailed identification of characteristics and primary purpose of apps [18]. Shen et al [19] and Bender et al [20] developed their own app characteristic coding schemes to meet the needs of their review focus that included functionality criteria assessment such as media type, user interface, and inclusion of tools to identify disease before symptom or signs. Previously, we have used the criteria proposed by the Institute for Healthcare Informatics specifically to assess app functionalities [12]. Reviews exist for identifying apps to support treatment of chronic diseases such as diabetes and [17] chronic pain [16,18] as well as for the prevention, detection, and management of cancer and [20] depression [19]. We have conducted prior reviews assessing the prevention of infectious diseases such as health care-associated infection prevention [21] and HIV [22]. However, no studies have assessed available apps to support TB prevention or treatment. To fill this gap, we conducted this systematic review to identify the TB prevention and/or treatment support-related apps available, report on their characteristics, and assess their functionalities. Our objectives were to (1) detect the number of TB-related apps available in the main app stores, (2) describe their characteristics, (3) evaluate their range of functionalities, (4) identify any rigorous testing of the available apps, and (5) scan the gray literature to identify if other TB-related apps are in progress.

## Methods

### Overview

Methods to identify, evaluate, and report findings for this study were guided by the Quality and Risk of Bias Checklist for Studies that Review Smartphone Applications [23] and the app functionality categories described in the Institute for Healthcare Informatics report [12]. The checklist comprises 8 criteria for evaluating and reporting the quality of smartphone health-related apps (eg, data collection time frame, clearly described app search strategy, concise description of the app appraisal methods). This checklist helped guide the search strategy and data characteristic extraction form development and ensure completeness of reporting of the results. The adapted app appraisal method, based on the app functionality categories from the Institute for Healthcare Informatics report, is described and defined below (see Table 1) [12].

### Search Strategy and App Selection

We conducted searches in 3 mobile app stores in the United States during June 2015: iTunes App Store, Google Play Store, and Amazon Appstore. We used the search terms *tuberculosis*, *TB*, *phthisis*, and *tuberculose*in each of the app stores. Apps were included if they focused on TB control efforts (eg, patient support, health care provider management, TB awareness) and excluded if they were not dedicated to TB efforts (eg, focused on other infectious diseases were games or unrelated) and not in English, Spanish, or Portuguese.

**Figure 1:**
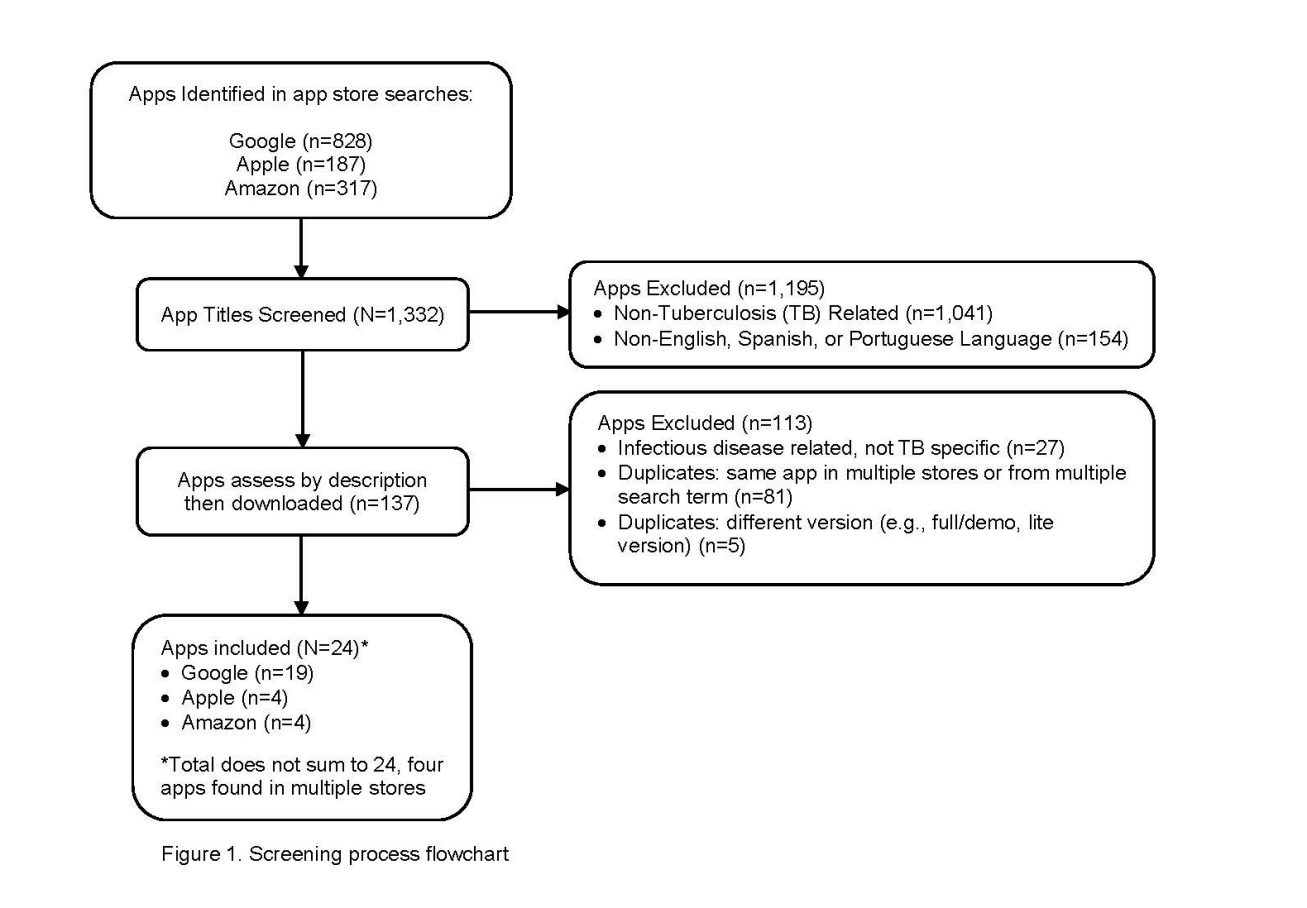
Flowchart.

Two team members, SI and LO, independently reviewed titles, full marketing descriptions, and screenshots of the potential apps for relevance and inclusion and discussed discrepancies until consensus was reached. All apps not meeting inclusion criteria were excluded. The list was then reviewed for duplicate apps identified from multiple searches and terms and “lite” or demo versions of apps to produce the final list of unique apps (see Figure 1 flowchart).

### Data Abstraction and App Functional Appraisal

Each eligible app was downloaded for functional appraisal. Emails were sent to listed company contacts for each of the apps requiring login and password to enter the full app content. We provided the purpose of this study and requested a test code, password, or further information about the app’s functionalities.

A data abstraction form (available upon request) was developed based on the app functionality criteria from the Institute for Healthcare Informatics report [12] and a prior app evaluation data abstraction process [21]. Each app was characterized for store availability (eg, Apple, Amazon), country developed, target end-users, cost to download, number of downloads, rating and number of reviewers contributing to the rating, date of last update, primary purpose, and any specific issues or features of apps (eg, audio function to read text, language options). The app functionalities were appraised based on the 7 functionality criteria and 4 functional subcategories (Table 1). Each app was assessed for having or not having each of the 11 functionalities and given a functionality score (0-11).

**Table 1:** App evaluation criteria.

Functionality	Definition
Inform	Provide information; can be in a variety of formats such as text, photo, or video
Instruct	Provide instructions to the user (eg, specific steps to take for TB test or diagnosis)
Record	Capture user-entered data and record functional subcategories Collect: enter and store health data on individual phone Share: transmit health data (eg, upload, transfer, email) Evaluate: evaluate the entered data Intervene: send alerts based on the data collected or propose behavioral interventions or changes (eg, alert to treatment provider regarding treatment adherence, alert user for TB dosage due)
Display	Display user-entered data graphically and provide an output (eg, report, medication log, contact screening results, search results)
Guide	Provide guidance based on user-entered information (eg, patient TB risk factor screening and recommendations for testing, medication dosage based on entered data - weight/age). Having the function to enter search terms to obtain information or diagnostic criteria was not considered a guide functionality
Remind/alert	Provide reminders to the user (eg, medication, follow-up appointments)
Communicate	Facilitate communication between providers, patients, consumers, caregivers, and medication administrators or provide links to social networks (eg, Facebook, email)

## Results

### Descriptive Characteristics

Search queries yielded 1332 potentially relevant apps, of which 24 were included in our review. Most of the apps were excluded because they were not TB-related. See Figure 1 flowchart for selection process and categories for exclusion. Multimedia Appendix 1 provides the full list of the included apps and their characteristics. All of the apps were free to download; however 7 were developed for specific settings (eg, countries and/or clinical or research sites) and had restricted access requiring a login and password. The target end-users for most apps (n=17) were health care providers (eg, frontline health care worker, clinician, or lab technician) while a few (n=4) were patient-focused or could be used by patients and providers (n=3). A total of 13 apps were rated by 2 to 40 reviewers, and the mean rating was 4.4 (range 3.7-5, SD 0.5) on a scale of 1-5. Of the 18 apps with a reported range of downloads, 11 had been downloaded less than 500 times.

### Functionality

**Figure 2:**
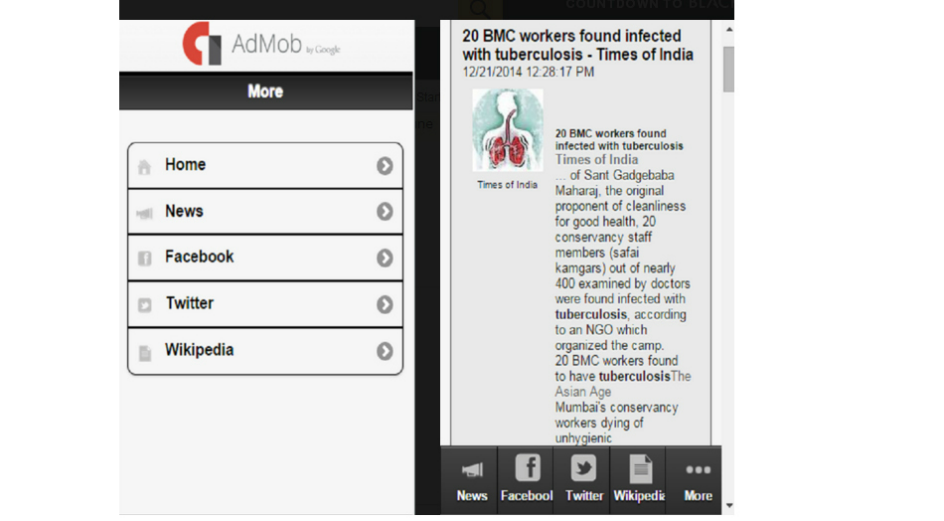
Tuberculosis News, TB Proof.

Multimedia Appendix 2 provides the included app functionalities and scores based on the 7 functionality criteria and 4 functional subcategories adapted from the Institute for Healthcare Informatics report. Most apps (n=17) had 4 or fewer functions out of 11 (range 1-6). The most common functionalities were inform and record (n=15). There were 6 apps with the functions to display, guide, remind/alert, or communicate. For those with the function to inform, the majority focused on providing information on TB diagnosis, treatment (eg, medication, regimen, and age considerations), and transmission. The 3 apps that had an alert/reminder function (eCompliance, eDetection, and MDR-TB MDR Clinic) were for health care workers to display reports of expected appointments or pending questionnaires needed during home visits. The function to communicate was restricted to access to a social media link such as Facebook page (Tuberculosis News, TB Proof) (Figure 2) or an email contact to report app problems (Explain TB, SNTC).

**Figure 3:**
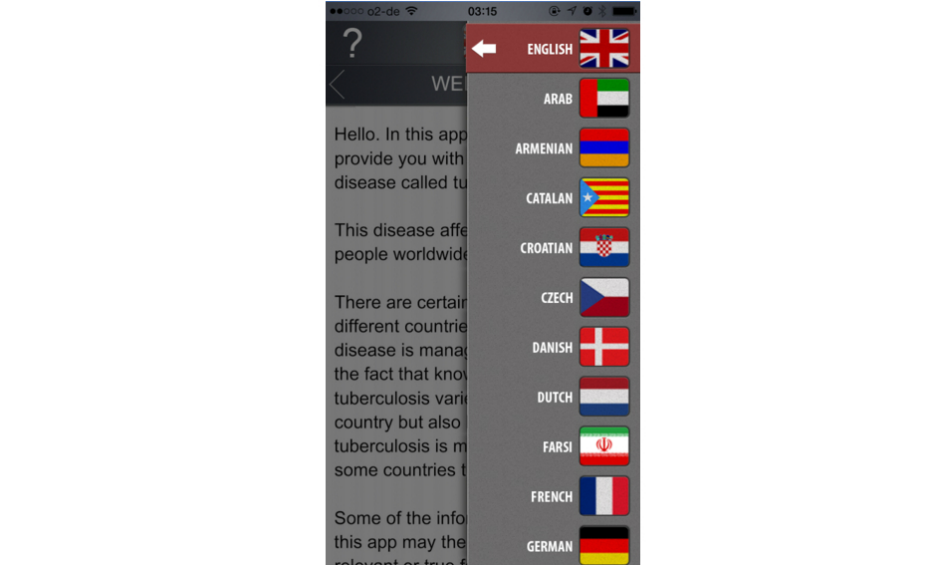
Explain TB.

The Explain TB app provided information in audio form and in multiple languages (Figure 3). One app did not function once downloaded (GuiaTB) and another crashed when opening a form within the app (eMocha). Seven of the apps require logins and passwords to access full functionality (eCompliance [Kenya, Jubilant Bhartia], eDetection, MDR-TB MDR Clinic Application, MDR-TB PHC Application, Global Fund TB, MINE TB, and TB REACH 4–Kotri).

Of the apps with the functionality to record, most collected (n=11) and shared data (n=10) (eg, sync to database or email results). Only 3 apps, CAD4TB, FIND TB, and TB Mobile, had the subfunction to evaluate (eg, provided feedback based on the data entered).

### Issues With Included Apps

Although a number of apps were identified as having various functionalities to support TB efforts, some had issues such as incorrect spelling and grammar, inconsistent responses to data entry, problems with crashing, or links to features that had no data.

**Figure 4:**
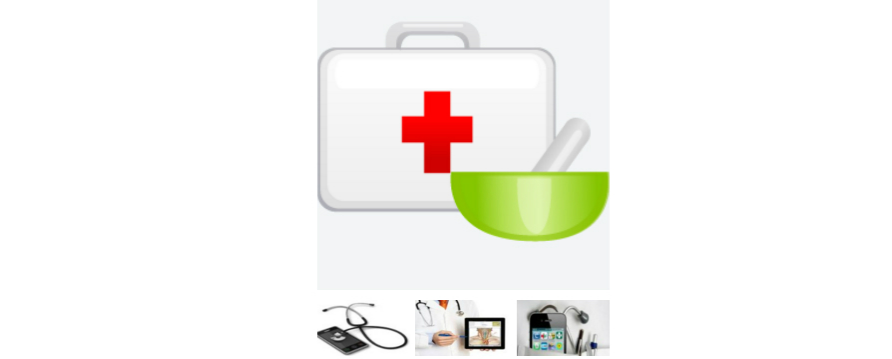
Tuberculosis Symptoms Guide.

For example, the Tuberculosis Symptoms Guide app (Figure 4) provides a TB syndrome screening form with 4 questions. The questions are as follows and are written verbatim from the app: Is the patient age 12 years? Is the patient HIV infected? Has the patient is in close contact with TB patients in home or at work? Does the patient has any symptoms? The recommended action provided after completing the screening questions is the same for all variations of responses (e.g., selecting “no” or “yes” to all questions). The response provided after answering the questions is: “The patient has systomps of TB he/she must be tested as soon as possible by a health care professional to determine the TB. If he/she is detected TB then the family members should also be checked for TB.” In addition, this app has nonfunctioning Contact Us and FAQ tabs, lists only the names of 5 TB drugs without further information, and has many words misspelled (eg, nigtht sweats, systomps of TB).

The eMOCHA TB Detect app has tabs for TB symptom screening, education (TB lectures, courses, and library), and a disclaimer. As of July 2015, there are video lectures on prevention and risks for acquiring TB and two tables in the library; however, there were no courses available. When selecting the TB symptom screening form, the app crashes and closes. This app was last updated in 2013 and may no longer be supported. Similarly, GuiaTB could be downloaded but crashed upon opening; it appears to no longer function or be supported.

**Figure 5:**
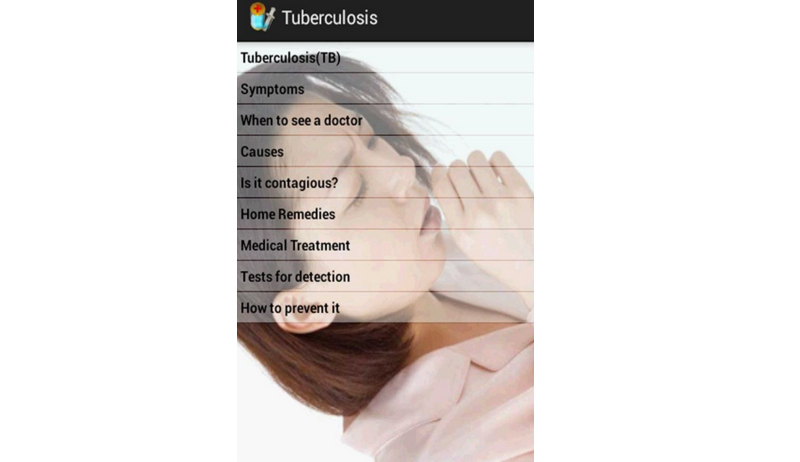
Tuberculosis (Amazon). Home remedies.

**Figure 6:**
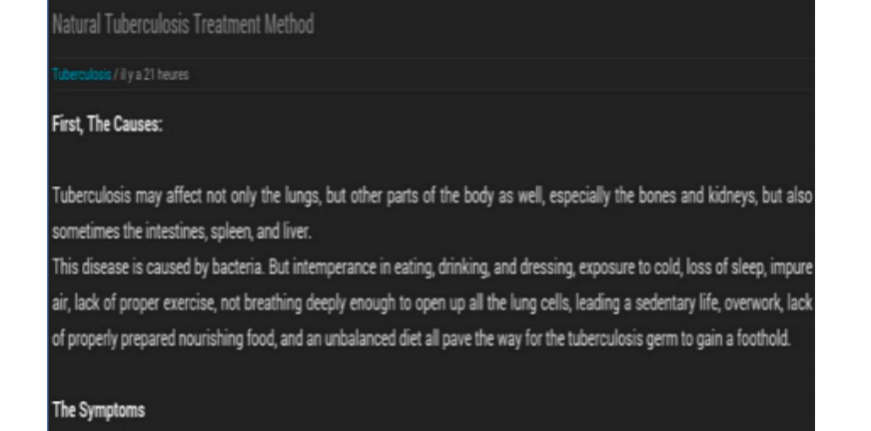
Tuberculosis (Google).

Of more concern were the apps that provided potentially harmful information. Tuberculosis (Amazon and Google) lists home remedy options or links to natural healers (Figures 5 and 6). For example, Tuberculosis (Google) provides links to information on medicinal herbs for treating TB and states “It seems like a cure all because it kills and neutralizes poisons;” these herbs are not consistent with evidence-based guidelines [24]. In addition, this app has many language options under settings but only the tabs and functions labels change language and not the content presented.

### Formal Research Studies on TB-Related Apps

Peer-reviewed publications were identified for only two of the included apps. A formal research study was conducted to evaluate the app CAD4TB. Researchers conducted a cohort study to assess the app’s sensitivity and specificity and found it helped accurately distinguish between the chest radiographs of culture-positive TB cases and controls [25]. Two publications described the development, features, and update of the TB Mobile app [26,27]. The researchers curated and prioritized data on molecules available in the Collaborative Drug Discovery database. App users can input molecular structures and search for potential targets to retrieve compounds known to be active. The authors state that the TB Mobile app has the potential to assist researchers in identifying targets for the development of antituberculosis drugs and purport that this mobile “cheminformatics” app could lower the barrier to drug discovery and promote collaboration among drug developers.

Another peer-reviewed publication reported on the design and piloting of a TB-related app not found in the app stores (Mobilize) [28]. Mobilize was developed to support health care workers in recording and tracking patients with multidrug-resistant TB. Last, the Centers for Disease Control and Prevention posted information on its webpage about the CDC LTBI app and its features [29] but there was no formal research study reported.

### TB-Related Apps in the Gray Literature

Three TB-related apps were identified in the gray literature (not found in the app stores) as in progress, being launched, or in testing (DOTsync, Nikshay, and an unnamed app to assist in contact tracing). DOTsync is described as replacing paper-based systems and providing community workers with a tool to document DOT, monitor infection control and drug complications, track nutritional support, and conduct TB contact tracing [30]. The DOTsync app was developed by FHI 360’s Control and Prevention of Tuberculosis project and the Myanmar Medical Association. Nikshay, launched by the government of India, aims to decrease the lengthy process of notifications, provide clinicians with a simpler case notification tool, and link the data to the Central Tuberculosis Division of the Ministry of Health and Family Welfare [31]. The aim of Nikshay is to increase enrollment in the national TB program, make the process easier for health care providers, and improve private sector reporting. The rollout started in March 2014 with email notification of the app being sent to general practitioners in Mumbai. The last app was unnamed but referred to in a poster abstract as an app developed to digitalize and automate contact tracing documentation in Botswana [32]. Preliminary results indicated that the app eliminated the need for manual data entry into the contact tracing database, required all form fields to be completed electronically prior to submission, automatically generated weekly and cumulative reports, and accurately captured the geographic coordinates of case homes. However, a final report was not identified in the literature.

## Discussion

### Principal Findings

The number of mHealth tools such as smartphone health-related apps has increased exponentially. As access to mobile phones, particularly smartphones and feature phones with the capacity to include mobile apps, continues to increase globally, the opportunity to harness their potential as a health-promoting tool grows as well. In conducting this research, we identified and evaluated functionalities of apps aimed at supporting TB prevention and treatment that were available in the leading mobile app stores. In addition, we identified apps reported in formal research studies and in the gray literature. Our review focuses on a specific, challenging disease and explores how apps available in the largest app stores might support TB prevention and treatment.

Almost all of the identified apps targeted health care providers as end-users, and the majority provided access to a broad spectrum of TB information or tools aimed to support these workers in monitoring, detecting, and documenting visits. Similarly, the apps identified in the gray literature were reported to support frontline health care workers in documentation of cases, contact tracing, or management of patients. One app was designed to support those aiming to identify and develop new TB treatment development options.

There was a clear lack of apps specifically designed to provide treatment support and care management for patients or promote their involvement in their treatment. The apps targeting patients as end-users provided information on TB disease, symptoms, diagnostics, treatment, and transmission. None of the apps were developed for patients to help them with their treatment regimen (eg, reminders/alerts for follow-ups). Opportunities have been recognized for mHealth and mobile apps to be developed for chronic and infectious diseases such as HIV, but apps for TB control have not been developed to the same extent [6].

Few TB-related apps were found to have undergone formal research evaluation. The app Mobilize (identified in the literature but not available in any of the app stores) was reported as feasible and effective at decreasing adverse events and improving communication; however, the authors report that app uptake was poor, and health care workers cited forgetfulness and a belief that patients should take more responsibility for their own care as reasons for not consistently using the app [28]. The authors recommend exploring the motivations of health care workers and technological enhancements prior to scaling up mHealth tools.

A few apps presented information that misinformed patients about appropriate TB treatment options. Misinformation in health care apps has been problematic in other areas (eg, Acne App claimed that its mobile app could cure acne by emitting colored light from a cell phone). The US Federal Trade Commission sued the marketer of this app claiming it was unfair and deceptive and provided information potentially harmful to patients. However, with more than 100,000 health-related apps estimated to be in production, it is difficult to regulate and oversee app content. There is a real concern that app users can be misinformed-especially given the lack of regulations. The US Food and Drug Administration in 2013 indicated that it will be overseeing apps that capture and transfer patient data [33,34]; however, the rest of the app content is left up to the developers. Currently, there are no specific regulations to assure reliability/validity of information.

Another issue was the lack of current support and/or functionality for some apps despite availability from an app store. In a prior review of HIV apps, we also found apps that no longer functioned. On the positive side, the apps we assessed requiring log-ins and passwords use OpenMRS [35] (an open source enterprise electronic medical record system) are supporting interoperability across programs and likely addressing issues of data security and privacy as recommended in the Principles for Digital Development [36].

### Implications

Existing mHealth tools can be improved and reused before new development is considered [36], but findings from this review suggest the need for development of apps for TB treatment for patients with the active disease. Apps that improve interaction with health care providers and support TB patient management and involvement in care appear to be lacking. Developing apps targeted at patients as end-users and involving TB patients in the design of these apps is recommended.

Although the checklist of 8 criteria for evaluating the quality and risk of bias for app evaluations is a helpful guide, its authors do not provide the criteria development process and indicate that the criteria were based on “simple facts and research transparency” [23]. In addition to the 8 criteria, we recommend specifying the last date updated because it is important to identify if the app continues to be managed/supported and is consistent with the most current clinical recommendations and guidelines. We also recommend including the country or setting for which the app was developed. The app characteristic evaluations have not been standardized. After we conducted our review, Stoyanov et al [37] published a mobile health app rating scale to classify and rate the quality of apps using 5 measures. In addition to app functionalities, the authors recommend review of patient engagement, aesthetics, and subjective quality. Our review assessed the range of downloads and user review ratings which may help inform the use and potential uptake. In addition, conducting searches outside of the app stores, such as in the gray literature, is helpful to identify apps under development or in production.

### Limitations

This review has a number of limitations. First, app stores have limited advanced searching capabilities; therefore, we may have missed potentially eligible apps. However, we conducted broad searches with multiple terms to capture any apps meeting our broad inclusion criteria. In addition, based on our gray literature search findings, there appear to be other TB-related apps being trialed that have not been released to the public or in the app stores. We describe the apps in development that we identified, but there may be others that were missed.

Second, we attempted to capture apps in 3 languages (English, Spanish, and Portuguese). However, only one app was identified in Portuguese and none were in Spanish. It is possible that, depending on the country from which the searches were conducted, the apps available at the app stores may vary and apps in these languages were not made available outside of their region, but it would be surprising to limit access to apps available in Spanish within the United States. Also there were apps in other languages that we had to exclude because we were unable to assess them.

Third, not all app stores provide the same data, and not all apps could be fully accessed. For example, the Amazon Appstore does not provide information on range of downloads or date of last update (only release dates are provided) and none of the apps had been rated or reviewed at time of this review. To overcome the limitation on accessing apps, we sent email inquiries to all of the app contacts that required login and password but did not receive a response from any of them. Therefore, for 7 apps we conducted the functionality assessments using the online screenshot images and marketing descriptions. The online screenshots contained from 2 to 7 screenshots for each app providing, in most cases, sufficient views to assess each functionality.

Last, although it was not the aim of our review, it is possible that there are apps for medication support that could provide medication management for patients on any treatment regimen, including TB. However, it is likely that they would not provide other TB-specific support such as information on infectivity, testing for close contacts, and common side effects of TB medications.

### Comparison With Prior Work

Prior reviews of apps were not TB-specific, did not include a functionality score, or did not search for apps in the gray literature [20,21,38-42]. An evaluation of HIV apps included an audit of formative patient-identified app function needs and preferences. Shen et al [19] evaluated apps based on their store description to provide an overall description of depression apps. De la Vega and Miro [16] could find no articles published on apps they identified in app stores for pain management but did find articles published on apps not available in app stores. They identified a gap between the scientific and commercial faces of mHealth.

### Conclusions

In our review, apps identified for TB efforts had minimal functionality, primarily targeted frontline health care workers, and focused on TB information (eg, general information, guidelines, and news) or data collection (eg, replace paper-based notification or tracking). Few apps were developed for use by patients and none were developed to support TB patient involvement and management in their care (eg, follow-up alerts/reminders, side effects monitoring) or to improve interaction with their health care providers, limiting the potential of these apps to facilitate patient-centered care. Given the complex challenges faced by patients with TB, there is a need for further app development targeting their needs. Our evaluation shows that more refined work is needed in this area. Involving TB patients in treatment in the design of these apps is recommended.

## Appendix

Multimedia Appendix 1 Characteristics of applications to support TB efforts.

Multimedia Appendix 2 App functionalities and scores.

## Abbreviations

DOT: directly observed therapy
mHealth: mobile health
TB: tuberculosis
WHO: World Health Organization
