# Supplementary material for: Smartphone Applications to Support Tuberculosis Prevention and Treatment: Review and Evaluation
Source: JMIR Mhealth Uhealth. 2016 May 13;4(2):e25. doi: 10.2196/mhealth.5022 (PMC4884267; doi:10.2196/mhealth.5022)
Supplement: Multimedia Appendix 1 [file mhealth_v4i2e25_app1.pdf]

App Name	Store	Country	Ratings (reviewers)	Download range	Target users	Last update	Description
Tuberculosis	Amazon	-	NR	-	Patient	08/02/13*	1-6 brief bullet points on 9 areas (TB definition, symptoms, causes, home remedies educational section
Tuberculosis Awareness	Amazon	-	NR	-	Patient	04/02/15*	Same information as Tuberculosis with different images
Tuberculosis News	Amazon	-	NR	-	Patient, Provider	12/21/14*	Links to: current TB news, The Global Fund to Fight AIDS, Tuberculosis and Malaria Facebook, Twitter, and Wikipedia TB page
Tuberculosis Symptoms Guide	Amazon	-	NR	-	Provider	02/25/14*	TB screening survey
CAD4TB	Apple	-	NR	-	Provider	05/28/15	Provides worksheet to simulate costs for screening and compare potential cases detected using digital chest X-ray with computer aided detection before testing for drug resistance (Xpert® MTB/RIF)
SNTC	Apple, Google	US (Univ. of Florida)	NR	-	Provider	08/21/14	Provides links to latest TB news, products (educational material in multiple languages), courses with GPS links to locations, and links to TB focused Webinars
CDC LTBI	Apple, Google	-	3.9 (16)	1000-5000	Provider	12/09/13	Guide for TB diagnosis, treatment, terms and abbreviations
Explain TB	Apple, Google	-	4.1 (10)	500-1000	Patient	03/10/14	Provides information about the disease, symptoms, diagnostics, treatment, transmission
TB Mobile	Apple, Google	-	3.9 (7)	500-1000	Provider	03/28/14	Database of molecules with activity against TB (chemical structures)
eCompliance (Jubilant)	Google	Kenya, Bhartia	3.7 (3)	50-100	Provider	04/30/15	Bio-metric enabled identification system to monitor and track TB patients
eDetection	Google	-	5.0 (2)	100-500	Provider	12/29/14	Supports contact tracing (identification and testing of people in contact with a person infected with TB).
eMOCHA TB Detect	Google	-	4.8 (4)	500-1,000	Provider	06/12/13	Assists in detecting TB and provides TB related education
Fight TB	Google	India	4.5 (40)	1000-5000	Provider	04/30/15	Quick reference to first line TB dosage calculator and treatment guidelines
FIND TB	Google		5.0 (5)	100-500	Provider	11/07/14	Helps accurately diagnose, provide information and TB management instructions
Global Fund TB	Google	South Africa	NR	10-50	Provider	06/02/15	Data collection tool to support TB screening and health record card.
GuiaTB	Google	Brazil	4.9 (7)	10-50	Provider	05/22/15	Rapid TB guide in Portuguese
MDR-TB Clinic App	Google	South Africa	NR	5-10	Provider	06/15/15	Developed for the MDR-TB Partnership. MDR-TB Clinic staff check in patients to the their appointment as they arrive
MDR-TB PHC App	Google	South Africa	NR	5-10	Provider	6/15/15	Developed for the MDR-TB Partnership. Helps enroll TB Suspects into clinics. Laboratory results update patient status
MINE TB	Google	South Africa	5.0 (2)	50-100	Provider **	05/28/15	Screening tool for those who work in South African mines and connects to OpenMRS server to store screening data.
TB Proof	Google	-	4.0 (2)	1000-5000	Patient, Provider	05/02/13	Survey to report TB related information on healthcare setting environments, problems, administrative control measures, type of personal protective equipment - TB Risk Report Form
TB REACH 4 – Kotri	Google	Pakistan	NR	10-50	Provider	04/16/15	Collect TB screening data
Tuberculosis	Google	-	NR	50-100	Patient, Provider	02/20/15	Provide links to TB articles on various topics and links to web pages.
Tuberculosis Information	Google	-	3.9 (14)	1000-5000	Patient	08/27/14	Provide TB disease information in 10 categories (e.g., causes, risk factors, symptoms, treatment and diagnosis).
TuberSpot	Google	-	4.5 (39)	500-1000	Provider	03/24/15	Game to identify TB bacilli in samples. Teaches shape, color, clusters, how to differentiate TB bacilli from artifact.

Note: – not specified, $ cost, NR not rated, * Release date, last update date not available. ** indicates that app is not a public app and targets to specific users.
