# Supplementary material for: Smartphone Applications to Support Tuberculosis Prevention and Treatment: Review and Evaluation
Source: JMIR Mhealth Uhealth. 2016 May 13;4(2):e25. doi: 10.2196/mhealth.5022 (PMC4884267; doi:10.2196/mhealth.5022)
Supplement: Multimedia Appendix 2 [file mhealth_v4i2e25_app2.pdf]

	Functionality							Record Subfunctionalities/Data Use				
**App Name**	**Inform**	**Instruct**	**Record**	**Display**	**Guide**	**Remind**	**Communicate**	**Collect**	**Share**	**Evaluate**	**Intervene**	Score
Tuberculosis	●											1
Tuberculosis Awareness	●											1
Tuberculosis News	●						●					2
Tuberculosis Symptoms Guide	●		●		●			●				4
CAD4TB			●	●				●		●		4
SNTC	●	●					●					3
CDC LTBI	●	●	●									3
Explain TB	●	●		●	●		●					5
TB Mobile	●		●	●					●	●		5
eCompliance (Kenya, Jubilant Bhartia)			●	●	●	●		●	●			6
eDetection			●	●		●		●	●			5
eMOCHA TB Detect	●	●	●					●	●			5
Fight TB	●	●			●							3
FIND TB	●	●	●		●					●		5
Global Fund TB			●		●			●	●			4
GuiaTB	●	●										2
MDR-TB Clinic App			●	●		●		●	●			5
MDR-TB PHC App			●					●	●			3
MINE TB		●	●	●				●	●			5
TB Proof			●				●	●	●			4
TB REACH 4 – Kotri			●					●	●			3
Tuberculosis	●											1
Tuberculosis Information	●	●										2
TuberSpot	●	●	●									3
	15	10	15	7	6	3	4	11	10	3	0	84
